# Bispecific Antibody Detection Using Antigen-Conjugated Synthetic Nucleic Acid Strands

**DOI:** 10.1021/acssensors.3c01717

**Published:** 2023-10-19

**Authors:** Davide Mariottini, Sara Bracaglia, Luca Barbero, Sebastian W. Fuchs, Christoph Saal, Sebastien Moniot, Christine Knuehl, Lorena Baranda, Simona Ranallo, Francesco Ricci

**Affiliations:** † Department of Chemical Science and Technologies, University of Rome Tor Vergata, Via della Ricerca Scientifica 1, 00133 Rome, Italy; ‡ RBM-Merck (an affiliate of Merck KGaA), Via Ribes 1, 10010 Turin, Italy; § Merck KGaA, Frankfurter Strasse 250, 64293 Darmstadt, Germany

**Keywords:** bispecific antibody, antibody detection, synthetic nucleic acid, DNA nanotechnology

## Abstract

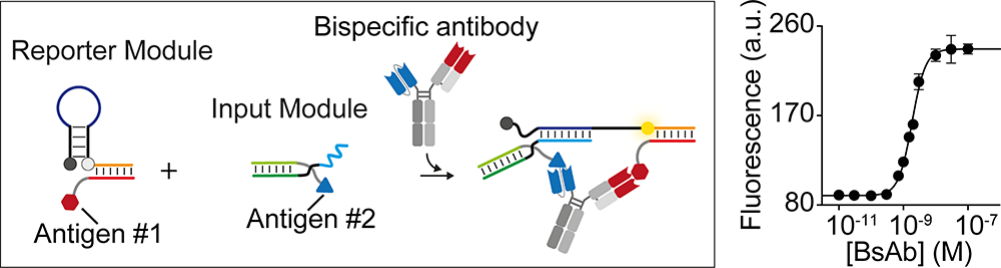

We report here the development of two different sensing strategies based on the use of antigen-conjugated nucleic acid strands for the detection of a bispecific antibody against the tumor-related proteins Mucin1 and epidermal growth factor receptor. Both approaches work well in serum samples (nanomolar sensitivity), show high specificity against the two monospecific antibodies, and are rapid. The results presented here demonstrate the versatility of DNA-based platforms for the detection of bispecific antibodies and could represent a versatile alternative to other more reagent-intensive and time-consuming analytical approaches.

Therapeutic antibodies have emerged over the past two decades as innovative drugs for the treatment of various diseases.^1−3^ Since the first Food and Drug Administration (FDA) approval of rituximab, a monoclonal antibody targeting the CD20 protein, more than 100 antibodies have been approved as drugs for the treatment of various diseases, such as cancer, autoimmune diseases, and chronic inflammation, showing significant response and long-term benefit.^4−7^ Advances in antibody technology and biology have recently led to the development of novel antibody formats to create therapeutic drugs with better efficacy.^1,8−11^ In this regard, bispecific antibodies (BsAbs) which, unlike monoclonal antibodies (mAbs), can recognize and block two distinct epitopes on the cell surface, represent the most promising direction for future cancer immunotherapy development.^12−14^ BsAbs are engineered immunoglobulins produced in vitro by biochemical, biological, or genetic processes.^15−19^ The bifunctional binding ability of BsAbs enables higher tumor specificity than mAb, ultimately leading to better therapeutic efficacy.^20−22^

New methods for detecting bispecific antibodies in clinical fluids are increasingly needed to characterize their pharmacokinetics and toxicokinetics and to find the best conditions for their efficacy.^23−26^ Current methods for detecting bispecific antibodies are mostly laboratory-based approaches used for structural and binding characterization of BsAbs. For example, several assays based on the adaptation of an enzyme-linked immunosorbent assay (ELISA) have been described.^27^ These assays use a “bridging” format in which one recombinant antigen is immobilized on a solid phase and a biotinylated version of the second antigen is added to form a ternary complex with the target antibody.^12,20,28−33^ This approach allows for highly sensitive BsAb detection but requires multiple washing and reaction steps, ultimately increasing the cost and reaction time. Recently, two surface-based approaches were also proposed. In one, a surface plasmon resonance (SPR)-based method that provides real-time information on the kinetics and affinity of the BsAb/antigen interaction in a label-free format was described.^28,31^ In another example, a chip with a Y-shaped DNA nanostructure labeled with two antigens and two optical dyes was used to characterize the binding properties of BsAb.^34^ While both systems provide excellent sensitivity to the target BsAb and appear to be suitable for characterizing BsAb binding, the complexity of the instrumentation required makes them less suitable for point-of-care detection.

In recent years, we and other research groups have reported several DNA-based devices that use synthetic antigen-conjugated strands for the optical detection of a wide range of monoclonal target antibodies.^35^ Despite the many advantages provided by these platforms (i.e., sensitivity, versatility, specificity, etc.), their possible use for the detection of bispecific antibodies has not yet been reported. Motivated by the above considerations, we demonstrate here the use of antigen-conjugated synthetic nucleic acid strands to develop two general platforms for the rapid, sensitive, inexpensive, and quantitative detection of BsAbs.

## Results and Discussion

**Figure 1:**
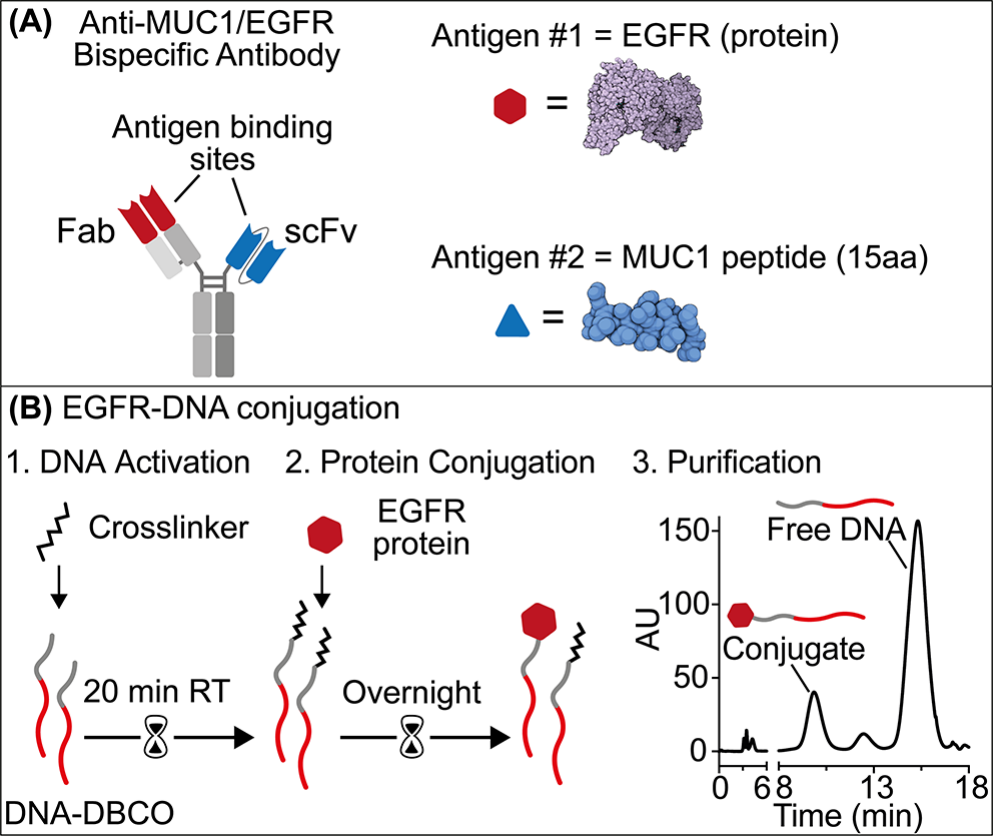
(A) General schematic of the bispecific Anti-MUC1/EGFR antibody. (B) Schematic representation of the reaction for conjugation of EGFR to a DNA strand and purification.

In this work we selected as a model BsAb an antibody that is engineered to recognize with one arm (Single-chain variable fragment, scFv) the tumor-associated Mucin1 (MUC1) protein and, with the second arm (Fragment antigen-binding, Fab), the epidermal growth factor receptor (EGFR) (Figure 1A).^14^ To develop a DNA-based platform for the detection of this antibody, we first need to conjugate the relevant antigens to two synthetic nucleic acid strands. First, we conjugated the human EGFRvIII protein to the 5′ end of a synthetic 27-nt dibenzocyclooctyne (DBCO)-modified DNA strand. To do so, we used a 1-ethyl-3-(3-dimethylaminopropyl)carbodiimide *N*-hydroxysuccinimide (EDC-NHS) ester coupling reaction (see also Supporting Information) followed by purification with ion exchange chromatography (Figure 1B). As antigen for the second binding site (targeting MUC1), we chose a 15 amino acid long exposed epitope recognized in tumor-associated MUC1 by Anti-MUC1 antibodies.^36^ Peptides are generally more difficult to conjugate to DNA strands and would lead to a more laborious purification of the conjugate. For this reason, we chose to conjugate the MUC1 peptide to a peptide nucleic acid (PNA) strand that, thanks to its pseudopeptide backbone, allows for easier conjugation while maintaining the same DNA sequencing ability. Specifically, the peptide residue at the N-terminus was conjugated to an 18-nt PNA strand by forming an amide bond by using a solid-phase synthesis method.

**Figure 2:**
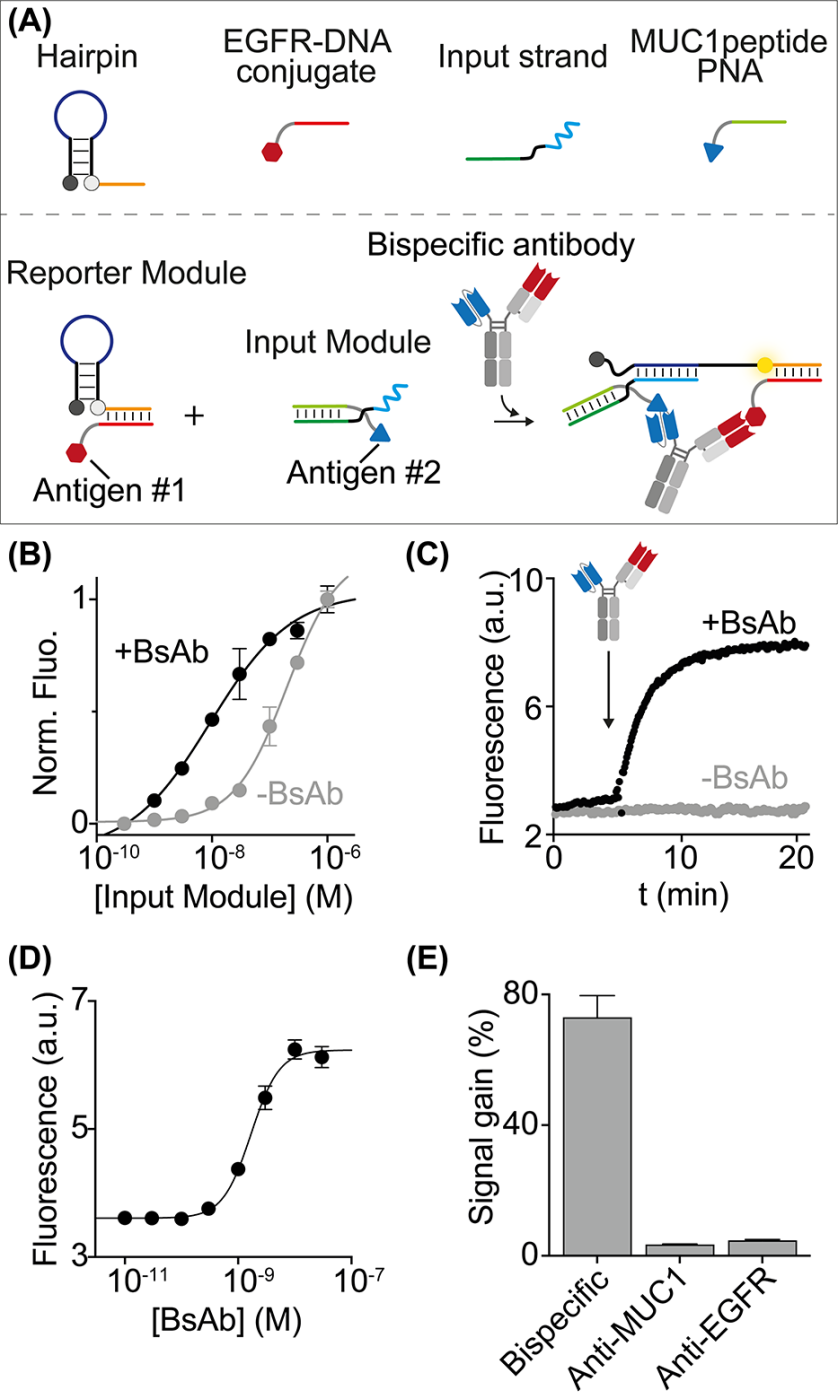
(A) Schematic of the antigen-conjugated nucleic acid strands platform. (B) Dose–response curve (fit eq (1)) obtained by adding an increasing concentration of the input module to a fixed (10 nM) concentration of the reporter module in the absence and presence of a saturating concentration (100 nM) of BsAb. (C) Fluorescence kinetic traces obtained in the absence (gray) and presence (black) of BsAb (100 nM) at a fixed concentration of reporter module (10 nM) and input module (30 nM). (D) Dose–response curve (fit eq (1)) at increasing concentration of BsAb. (E) Signal gain values (fit eq (3)) obtained at a saturating concentration (100 nM) of the BsAb and the two related monoclonal antibodies. Experiments were performed in 20 μL of 10 mM Na_2_HPO_4_, 137 mM NaCl, and 2.7 mM KCl at pH 7.4 at 25 °C. Experimental values in this and the following figures represent averages of three separate measurements, and error bars reflect standard deviations.

Using the antigen-conjugated nucleic acid strands described above, we set out to demonstrate two possible strategies for BsAb detection. In the first one we adapted an approach recently described by our group for monoclonal antibody detection based on antibody-induced colocalization of antigen-conjugated nucleic acid strands.^37^ The system comprises two modules: a reporter module and an input module. The reporter module is a duplex DNA obtained by hybridization of a fluorophore/quencher-modified hairpin DNA strand flanking a 15-base single strand and the EGFR-conjugated DNA strand described above (Figure 2A). The input module is instead a duplex of a DNA strand with a portion complementary to the loop of the hairpin DNA strand and the MUC1 peptide-conjugated PNA strand (Figure 2A). Bivalent binding of BsAb to the antigen-conjugated strands colocalizes the reporter and input modules, thus increasing their local concentration and enabling their hybridization (Figure 2A). Such antibody-induced hybridization triggers the opening of the stem-loop structure and enhances the fluorescence as a function of the antibody concentration.

To optimize the platform’s performance for BsAb detection and maximize its signal response, we first generated binding curves by adding increasing concentrations of the input module at a fixed concentration (10 nM) of the reporter module in the absence and presence of a saturating concentration of BsAb (100 nM). In the presence of the target antibody, we observe an increase in binding affinity (*K*_1/2_ (−BsAb) = 195 ± 2 nM and *K*_1/2_ (+BsAb) = 9.4 ± 0.4 nM), supporting the hypothesis that antibody-induced colocalization is critical for the sensing mechanism (Figure 2B). We find that a concentration of 30 nM of the input module leads to the largest difference in fluorescence signal between the absence and presence of BsAb (Figure S1). Using the optimized concentrations of the reporter module (i.e., 30 nM) and the input module (i.e., 10 nM), we tested the platform to detect the BsAb. Since it is a direct approach, no washing or multiple reaction steps are required, and we observe signal saturation after addition of the BsAb after approximately 30 min (Figure 2C). The platform proves to be sensitive (*K*_1/2_ = 1.7 ± 0.4 nM) with a calculated detection limit (defined here as the antibody concentration that gives a signal equal to the blank value plus three standard deviation) of 0.6 nM (Figure 2D). The presence of the two related monoclonal antibodies causes only a minimal signal (2.7% for Anti-MUC1 and 3.3% for Anti-EGFR) (Figure 2E).

Comparable results in terms of sensitivity and specificity were obtained with platforms designed for the detection of the two monoclonal antibodies using the same recognition element in the two modules (Figures S2 & S3).

**Figure 3:**
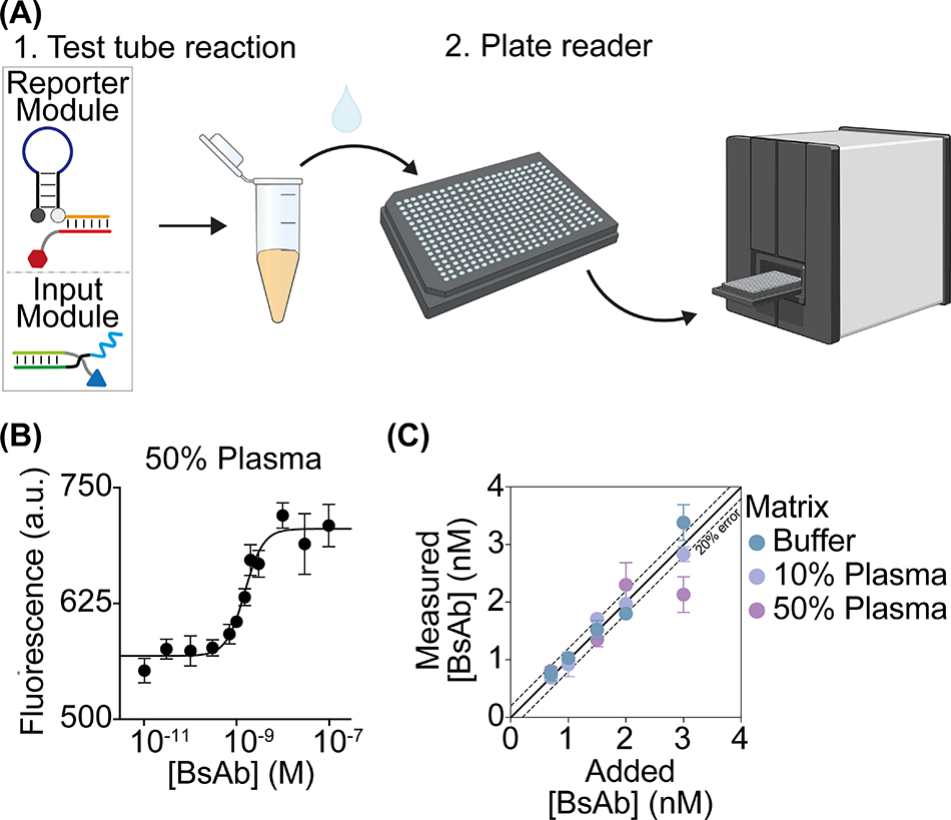
(A) Schematic representation of the plate reader platform for BsAb detection. (B) Fluorescence signals in a 50% plasma solution spiked with increasing concentrations of BsAb (fit eq (1)). (C) Correlation between added (0.7, 1, 1.5, 2, 3 nM) and measured BsAb concentrations (fit eq (4)) in different matrix samples (buffer solution, 10% plasma, and 50% plasma). Experiments were performed in a 20 μL solution containing 10 mM Na_2_HPO_4_, 137 mM NaCl, and 2.7 mM KCl, pH 7.4, containing the reporter module (10 nM), the input module (30 nM), and the BsAb at the indicated concentration.

To demonstrate the potential application of our sensing platform at the point-of-care, we adapted the antibody detection measurements to the format of a plate reader (Figure 3A). Using the plate reader format, the platform confirmed sensitive detection of BsAb directly in a 50% plasma sample (*K*_1/2_ = 1.5 ± 0.2 nM; limit of detection (LOD) = 0.4 nM) (Figure 3B). The analytical performance of our platform was evaluated by spiking different matrix samples (buffer solution, 10% plasma, and 50% plasma) with five known BsAb concentrations (0.7, 1, 1.5, 2, 3 nM; *n* = 3 for each concentration) during intrarun experiments. A BIAS% (or systematic error), defined as the difference between the expected result and the true value, less than +15% was obtained with these experiments. A good correlation (within ±20% error) between spiked and measured BsAb concentration in the linear range was also observed (Figures 3C & S4). The CV% (Percent Coefficient of Variation), defined as the agreement between independent measurements and the precision obtained by our method, was <3% (see also Supporting Information for analytical characterization). Finally, the lowest tested BsAb concentration determined with acceptable accuracy and precision, defined as the Low Limit of Quantification (LLOQ), was 0.7 nM.

We also evaluated the stability of the method by calculating the percent stability (STAB%), defined as the ratio between the measured concentration and the added concentration after storage of the platform under different conditions. Short-term (or benchtop) stability was evaluated by testing the BsAb concentrations of the linear range after the platform components were stored at room temperature (RT) for 4 h. Instead, freeze–thaw stability was evaluated by testing these concentrations after the components were stored at −80 °C for at least 12 h and then thawed three times. The results showed that the method is stable with an STAB% between 80 and 120%.

**Figure 4:**
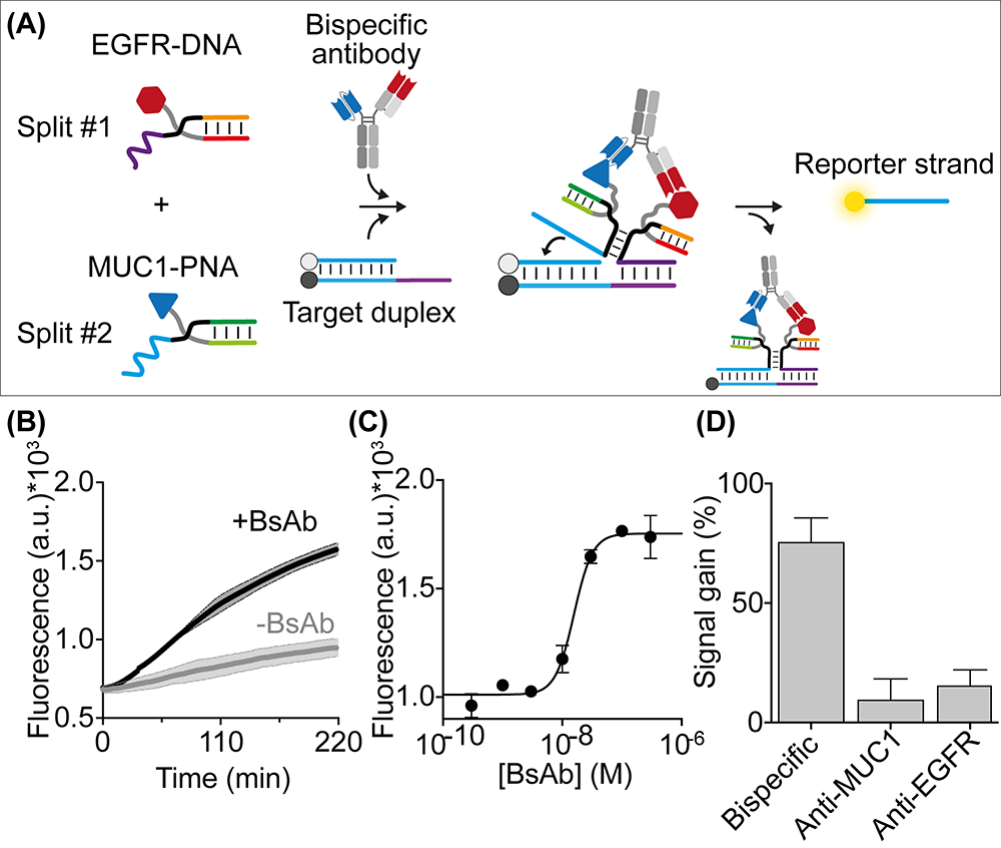
(A) Antibody-responsive strand displacement reaction for the detection of BsAb. (B) Fluorescence kinetic traces obtained in the absence (gray) and presence (black) of BsAb. (C) Fluorescence values in a 50% plasma solution supplemented with increasing BsAb concentrations (fit eq (1)). (D) Signal gain values (fit eq (3)) at a saturating concentration (100 nM) of BsAb and the two related monoclonal antibodies. Experiments were performed in 20 μL of 50 mM Na_2_HPO_4_, 150 mM NaCl, pH 7.0 containing the DNA target duplex (60 nM), split #1 + #2 (both at 100 nM), EGFR-DNA conjugate and MUC1-PNA conjugate (both at 120 nM), and BsAb at the indicated concentration.

Because antigen-conjugated synthetic nucleic acid strands are programmable, they can be used to detect BsAb via a variety of mechanisms. To demonstrate this, we employed a second strategy for the detection of the same bispecific target antibody. The approach employs an antibody-induced strand displacement reaction previously demonstrated for monospecific antibodies.^38,39^ Specifically, this strategy uses a target duplex labeled with a fluorophore/quencher pair and two unmodified scaffold strands (split #1 and #2, Figure 4A) that can hybridize to the antigen-conjugated DNA strands. The scaffold strands consist of three sections: (i) a complementary sequence to the MUC1 peptide-PNA or EGFR-DNA conjugates; (ii) a stem-forming section (black); and (iii) a toehold or invasion sequence required to activate the strand displacement reaction. Binding of BsAb to the two recognition elements induces colocalization of the bimolecular complexes (split #1/EGFR-DNA conjugate and split #2/MUC1 peptide-PNA conjugate) and promotes hybridization between the stem-forming portions leading to activation of the strand displacement reaction. This induces the release of the fluorophore-labeled reporter strand and a subsequent increase in the measured fluorescence signal. This strategy could, in principle, allow one to reduce possible non-specific interactions in absence of the target antibody, enabling a better optimization of the signal-to-noise ratio.

As a first step toward the characterization of the Ab-induced colocalization, we designed a bivalent DNA strand that acts as an Ab mimic and binds the first portion of Split #1 and #2, inducing a similar colocalization to that expected from the binding of a bivalent antibody (Figure S5). We then used our platform to detect the BsAb in a 50% plasma solution. The presence of the antibody efficiently induces a strand displacement reaction in a concentration-dependent manner (*K*_1/2_ = 15 ± 1 nM; LOD = 8 nM) (Figure 4B,C). The overall reaction efficiency increases with BsAb concentration until it saturates at about 100 nM. BsAb detection is highly specific, as no significant fluorescence signals are observed at saturating concentrations of the related monoclonal antibodies (Anti-MUC1 and Anti-EGFR antibodies) (Figure 4D).

## Conclusions

In the present study, we have demonstrated two different sensing platforms that employ antigen-conjugated nucleic acid strands for the detection of a bispecific antibody against the tumor-related proteins Mucin1 and EGFR. The systems we developed can efficiently detect the target BsAb with high sensitivity, specificity (no significant activation with monospecific antibodies was observed), and good selectivity in complex sample matrices (plasma). They also present advantageous features in comparison with standard methods such as ELISA that make them suitable for point-of-care applications. In particular, both platforms developed in this work, as also other DNA-based systems for antibodies detection reported recently by our and other research groups,^35^ are rapid and cost-effective and can be easily adapted to detect other therapeutic bispecific antibodies.^40^ For a more detailed comparison between our approaches, other DNA-based sensors, and ELISA we refer to our recent Perspective published in this journal.^35^

The development of similar DNA-based point-of-care methods for the detection of therapeutic bispecific antibodies would improve the characterization and monitoring of immunotherapies, thereby increasing their efficacy. A possible limitation of the approaches we described in this work is that, like the majority of analytical systems that do not rely on an amplification step, they cannot achieve the sensitivity of other amplification-based antibody detection methods such as ELISA.^33^ For example, in this work we achieve sensitivities in the low nanomolar level, which is in the same order of the plasma level expected in patients treated with therapeutic antibodies.^41^ But for applications in which the level of the target is expected to be below the nanomolar sensitivity reached here, an additional amplification step should be added. This could be achieved through, for example, the use of enzymes or nonenzymatic reaction cascades.^42,43^

## Supporting Information Available

Reagents and materials, oligonucleotide sequences, experimental details; Kinetic traces, binding curves, and specificity experiments with Anti-MUC1 and Anti-EGFR monoclonal antibodies; Binding curves in buffer, 10% and 50% plasma samples with Bispecific antibody; Binding curve with Ab mimic strand (PDF)
