# Supplementary material for: Bispecific Antibody Detection Using Antigen-Conjugated Synthetic Nucleic Acid Strands
Source: ACS Sens. 2023 Oct 19;8(11):4014–9. doi: 10.1021/acssensors.3c01717 (PMC10683503; doi:10.1021/acssensors.3c01717)
Supplement: Supplementary file 1 — se3c01717_si_001.pdf [file se3c01717_si_001.pdf]

Supplementary information:

Bispecific antibody detection using antigen-conjugated synthetic nucleic-acid strands

Davide Mariottini,[a,‡] Sara Bracaglia,[a,‡] Luca Barbero,[b] Sebastian W. Fuchs,[c] Christoph Saal,[c,†] Sebastien Moniot,[c] Christine Knuehl,[c] Lorena Baranda,[a] Simona Ranallo,[a,*] Francesco Ricci[a,*]

[a] Department of Chemical Science and Technologies, University of Rome Tor Vergata, Via della Ricerca Scientifica 1, 00133, Rome, Italy

[b] RBM-Merck (an affiliate of Merck KGaA), Via Ribes 1, 10010, Turin, Italy

[c] Merck KGaA, Frankfurter Strasse 250, 64293, Darmstadt, Germany

*Email: simona.ranallo@uniroma2.it; francesco.ricci@uniroma2.it

‡ These authors contributed equally

† Boehringer Ingelheim Pharma GmbH & Co. KG, Birkendorfer Str. 65, 88400, Biberach an der Riß, Germany

Materials

Chemicals

Reagent-grade chemicals (Sodium Chloride, Disodium Hydrogen Phosphate Phosphate-buffered saline (PBS tablets)) were purchased from Sigma-Aldrich (St Louis, Missouri) and used without further purifications. Anti-MUC1 monoclonal antibody, Anti-EGFR and Anti-MUC1/EGFR bispecific antibody were kindly provided by Merck (Darmstadt, Germany). The antibodies were aliquoted and stored at 4 °C or at -20°C for immediate use and at -80 °C for long-term storage.

DNA Sequences

HPLC purified oligonucleotides were purchased from Biosearch Technologies (Risskov, Denmark), Metabion International AG (Planegg, Germany) and Biomers GmbH (Ulm/Donau, Germany). PNA/Peptide chimera probes were purchased from Panagene (South Korea). All sequences were designed using Nupack or IDT oligoanalyzer tools.[1,2]

1) Anti-MUC antibody detection platform

Name	Sequence
Reporter strand	5’- **AGA ATA AAA CGC CAC TG** T(6Fam) *ACG TG* ATC TAA TGG TGA GTC *CAC GT* T(BHQ1) -3’
Input Strand	5'- AGA ATA AAA CGC CAC TG T TTT TTT TTT TTT TTT TTT TTT TTT GAC TCA CCA TTA G – 3’
MUC1-PNA strand	5’- CAG TGG CGT TTT ATT CT -3’ N_{term}- (APDTRPAPGSTAPPA)-C_{term}

Here in the reporter strand sequence the *italic* bases denote the stem forming portion, the underlined bases the loop portion and **bold** bases the anchoring portion for the input strand. In the MUC1-PNA strand the sequence in parentheses represents the selected peptide epitope portions that is terminally conjugated to a PNA strand.

2) Anti-EGFR antibody detection platform

**Name**	**Sequence**
Reporter strand	5'- (BHQ1)T *TGC AC* <u>CTG AGT GGT AAT CTA</u> *GTG CA* T(6FAM) T **AG AAT AAA ACG CCA CTG** -3'
Input Strand	5'- G ATT ACC ACT CAG TTT TTT TTT TTT TTT TTT TTT TTT T AGA ATA AAA CGC CAC TG -3'
EGFR-DNA Conjugate	5'- (EGFR)- C6 Amino TTT TTT TTT T CAG TGG CGT TTT ATT CT -3'

Here in the reporter strand sequence the *italic* bases denote the stem forming portion, the <u>underlined</u> bases the loop portion and **bold** bases the anchoring portion for the input strand.

3) Bispecific antibody detection platform

**Name**	**Sequence**
Reporter strand	5'- (BHQ1)T *TGC AC* <u>CTG AGT GGT AAT CTA</u> *GTG CA* T(6FAM) **TAG AAT AAA ACG CCA CTG** -3'
Input Strand	5'- G ATT ACC ACT CAG TTT TTT TTT TTT TTT TTT TTT TTT T GGA TAG TCG AAT TTA GT -3'
EGFR-DNA Conjugate	5'- (EGFR) C6 Amino TTT TTT TTT T CAG TGG CGT TTT ATT CT -3'
MUC1-PNA strand	5'- ACT AAA TTC GAC TAT CC -3' N_{term}- (APDTRPAPGSTAPPA) - C_{term}

Here in the reporter strand sequence the *italic* bases denote the stem forming portion, the <u>underlined</u> bases the loop portion and **bold** bases the anchoring portion for the input strand.

4) Strand Displacement-based antibody detection platform

**Name**	**Sequence**
Target duplex strand#1	5'- (BHQ2) - <u>CCT CAT CAT CAT ATA CGT CAC</u> **CTA TCC CAT TCT -**3'

Target duplex Reporter Strand	5'- GTG ACG TAT ATG ATG ATG AGG – (Cy3) -3'
Split#1 Scaffold	5'- **AGA ATG GGA TAG** – TT – *GTC TGC* – AGA ATA AAA CGC CAC TG -3'
Split#2 Scaffold	5'- GGA TAG TCG AAT TTA GT – *GCA GAC* – TT – GTG ACG TAT ATG ATG ATG AGG -3'
Ab mimic	5'- CAG TGG CGT TTT ATT CT TTT TTT TTT TTT TTT ACT AAA TTC GAC TAT CC -3'
EGFR-DNA Conjugate	5'- (EGFR)-C6 Amino TTT TTT TTT T CAG TGG CGT TTT ATT CT -3'
MUC1 PNA Chimera	5'- ACT AAA TTC GAC TAT CC -3' N_{term}- (APDTRPAPGSTAPPA) - C_{term}

Here the *italic* bases in the Split#1 and #2 sequences denote the stem forming portion, the underlined sequences represent the invading portion, while the **bold** bases denote the toehold domain. The sequence in parentheses represents the selected peptide epitope portions that is terminally conjugated to PNA.

Methods

EGFR-DNA conjugation

EGFR protein is covalently conjugated to a DNA strand using the Amine Coupling Kit 3 according to the manufacturer's instructions (Dynamic Biosensors). All these kit reagents (i.e., crosslinker, spin desalting column, etc.) have been optimize to maximize the conjugation yield. Specifically, EGFR protein tablets were diluted in PBS buffer (10 mM Na_2HPO_4, 137 mM NaCl, 2.7 mM KCl, pH 7.4) to 1 µg/µL and stored at 20°C. The DNA strand modified with a DBCO group at one end is mixed with a crosslinker solution and the excess of crosslinker is removed with a Spin Desalting Column. EGFR protein is added to the solution for an incubation of 1 h at RT and then overnight at 4 °C. Subsequently, the conjugate is purified from unreacted oligos by ion exchange chromatography using a proFIRE device (Dynamic Biosensors, Planegg, Germany) according to the manufacturer's instructions. The ion-exchange column is pre-equilibrated with buffer A (50 mM Na_2HPO_4/NaH_2PO_4, 150 mM NaCl, pH 7.2) before the injection of 160µL of EGFR-DNA conjugate. The conjugate is eluted with a salt gradient using buffer B (50 mM Na_2HPO_4,/NaH_2PO_4, 1 M NaCl, pH 7.2) with a flow rate of 1 mL/min. After purification, buffer exchange of the conjugate is performed using Centrifugal Filter Units with 3 kDa molecular weight cut-off (MWCO) against 10 mM Na_2HPO_4, 137 mM NaCl, 2.7 mM KCl, pH7 7.4. Conjugate concentration is finally determined by measuring the absorbance at 260 nm.

Fluorescence experiments

Fluorescent experiments were conducted in 45 µL 10 mM Na_2HPO_4, 137 mM NaCl, 2.7 mM KCl, pH 7.4 at 25°C. Equilibrium fluorescence measurements were obtained using a Cary Eclipse Fluorimeter (Varian) respectively with excitation at 490 (± 5) nm and acquisition at 520 (± 5) nm (Figure 2, S1-3). Fluorescence experiments adapted to the plate reader format were carried out in 20 µL 10 mM Na_2HPO_4, 137 mM NaCl, 2.7 mM KCl, pH 7.4 at 25°C using Tecan Infinite 200 Pro with top reading mode and black, flat bottom non-binding 384 well plates. Fluorescence signals were collected respectively with excitation at 488 (± 9) nm and acquisition at 523 (± 20) nm (Figure 3, S4). Strand displacement reactions were conducted in 20 µL 50 mM NaH_2PO_4, 150 mM NaCl, pH 7.0 at 25°C using the plate reader format (Figure 4, S5) respectively with excitation at 646 (± 9) nm and acquisition at 680 (± 20) nm.

Data analysis

The experimental values represent averages of three separate measurements and the error bars reflect the standard deviations. Binding curves were fitted with the following four parameter logistic equation:

$$F = F_{min} + (F_{max} - F_{min})\frac{[\text{Target}]^{nH}}{{K_{1/2}}^{nH} + [\text{Target}]^{nH}} \quad (1)$$

where, F_{min} and F_{max} are the minimum and maximum fluorescence values, $K_{1/2}$ is the equilibrium antibody concentration at half-maximum signal, n_H is the Hill coefficient, and [Target] is the concentration of the specific antibody added.

For a more ready interpretation of the results, normalization has been obtained on a 0−1 scale using the following formula:

$$\text{Norm. Fluo.} = \frac{[F_T - F_0]}{[F_{max} - F_0]} \quad (2)$$

where F_T is the fluorescence signal obtained in presence of the target antibody, F_0 is the fluorescence signal obtained in the absence of target and F_{max} represents the maximum fluorescence signal of the platform at a saturating concentration of target (Rel. Fluor. = 1).

Signal Gain (%) values are calculated as the relative signal change registered upon the addition of saturating concentration of target antibodies (or non-specific antibodies) using the following formula:

$$\text{Signal gain (\%)} = \frac{F_{\text{target}} - F_0}{F_0} \times 100 \quad (3)$$

where F_{target} is the signal in the presence of different concentration of the target; F_0 is the background signal.

Accuracy tests were performed by adding known amounts of Bispecific antibody into either PBS and blank 10% or 50% plasma samples. Bispecific antibody quantification ([Target]) was performed by using the following formula:

$$[\text{Target}] = K_{1/2}\left(\frac{(F_{max} - F_{min})}{(F_{target} - F_{min})} - 1\right)^{1/nH} \quad (4)$$

F_{min} and F_{max} are the minimum and maximum fluorescence values, $K_{1/2}$ is the equilibrium antibody concentration at half-maximum signal, n_H is the Hill coefficient extrapolated from the binding curves in Figure S4. The Percent Coefficient of Variation (CV %) and the Percent Relative Error (BIAS %) of the estimated concentration were calculated using the following formulas:

$$\text{CV \%} = \frac{\text{St. Deviation}}{\text{Mean Value}} \times 100 \quad (5)$$

$$\text{BIAS \%} = \frac{\text{Measured Conc.} - \text{Added Conc.}}{\text{Added Conc.}} \times 100 \quad (6)$$

The Percent Stability (STAB %) of the method was assessed after keeping the reaction components at room temperature (RT) for 4h or after three cycle of freeze-thaw and was calculated using the following formula:

$$\mathrm{STAB}\ \% = \frac{\text{Average Measured Conc.}}{\text{Added Conc.}} \times 100 \qquad (7)$$

The limit of detection (LOD) was determined as the concentration that reaches three standard deviations above a blank signal, instead the low limit of quantitation (LLOQ) is the lowest concentration that can be quantified in a reliable way, meeting BIAS% and CV% criteria.

Supplementary Figures

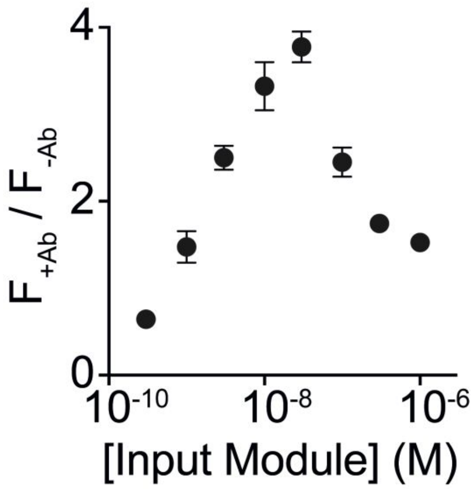

Figure S1 Plot showing the ratio between the fluorescence signals of the binding curves reported in Figure 2B and obtained in 20 μL buffer solution (10 mM Na_2HPO_4, 137 mM NaCl, 2.7 mM KCl, pH 7.4) containing the reporter module (10 nM) and in the presence ($F_{(+Ab)}$) (100 nM) and absence ($F_{(-Ab)}$) of the BsAb at increasing concentration of the input module. The fluorescence signals were measured at 520 nm after 30 min of incubation.

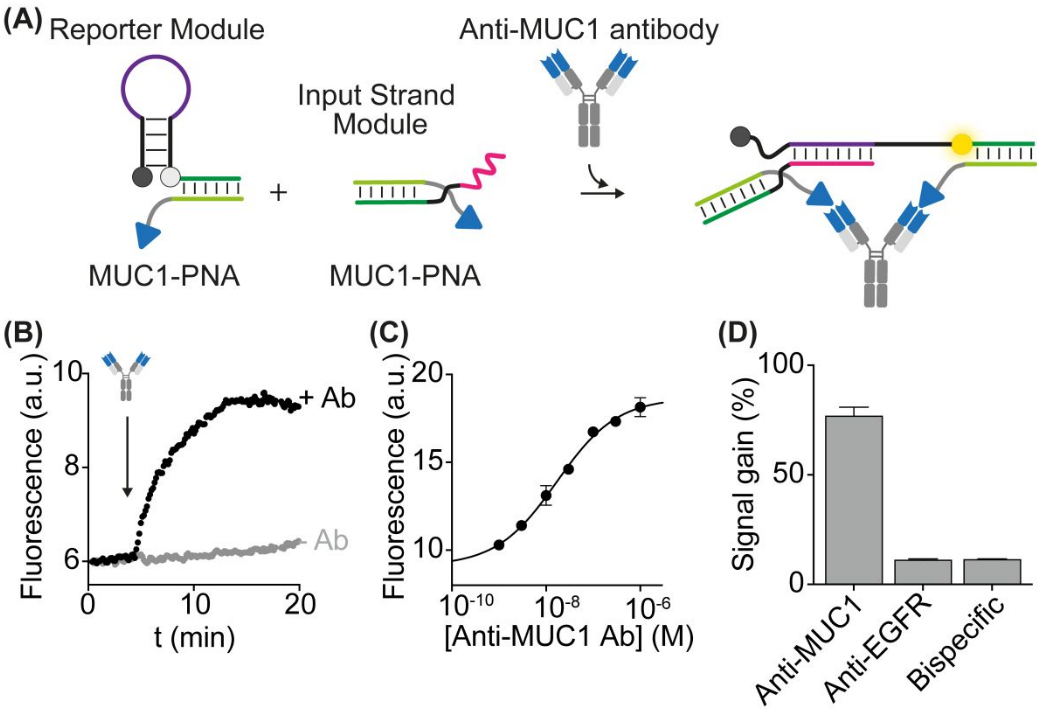

Figure S2 (A) General scheme of the antigen-conjugated nucleic acid strands platform for Anti-MUC1 antibody detection. (B) Fluorescence kinetic traces in the presence (100 nM) and absence of Anti-MUC1 antibodies. (C) End point values obtained at increasing concentration of Anti-MUC1 antibodies (fit eq. (1)). (D) Signal gain values (fit eq. (3)) observed at saturating concentration (100 nM) of Anti-MUC1 antibody, and with non-specific antibodies. The experiments were performed in a 20 µL buffer solution (10 mM Na_2HPO_4, 137 mM NaCl, 2.7 mM KCl, pH 7.4) at 25°C containing the reporter module (10 nM), the input module (30 nM) and antibodies as indicated. The fluorescence signals were measured at 520 nm after 30 min of incubation.

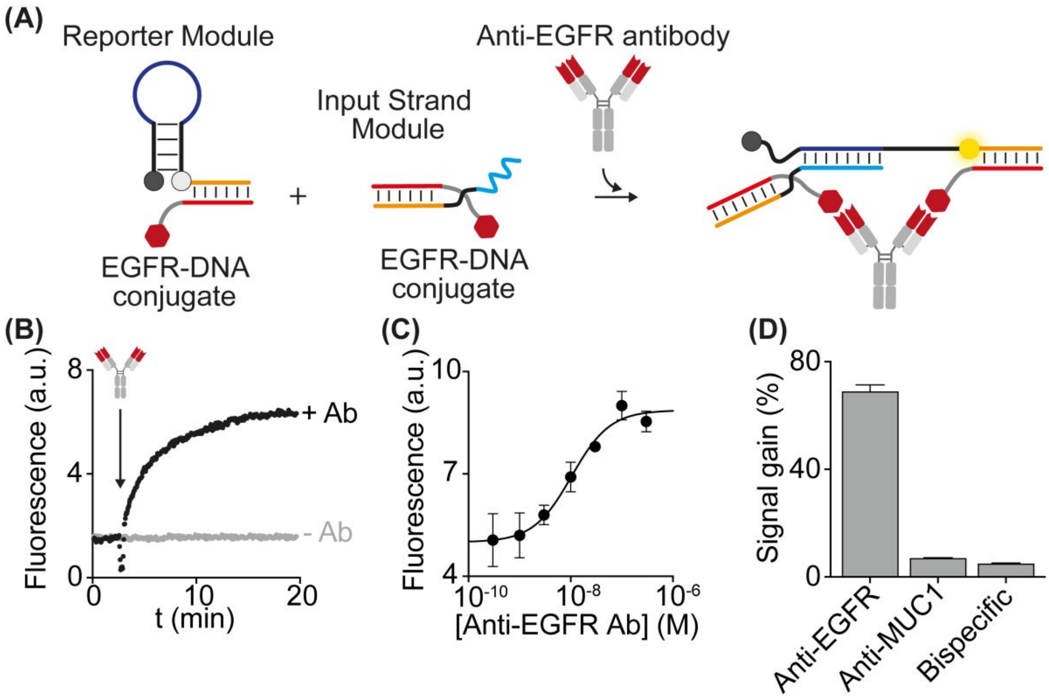

Figure S3 (A) General scheme of the antigen-conjugated nucleic acid strands platform for Anti-EGFR antibody detection. (B) Fluorescence kinetic traces in the presence (100 nM) and absence of Anti-EGFR antibodies . (C) Dose-response curve at increasing concentration of Anti-EGFR antibodies (fit eq. (1)). (D) Signal gain values (fit eq. (3)) observed at saturating concentration (100 nM) of Anti-EGFR antibody, and with non-specific antibodies. The experiments were performed in a 20 µL buffer solution (10 mM Na_2HPO_4, 137 mM NaCl, 2.7 mM KCl, pH 7.4) at 25°C containing the reporter module (10 nM), the input module (30 nM) and antibodies as indicated. The fluorescence signals were measured at 520 nm after 30 min of incubation.

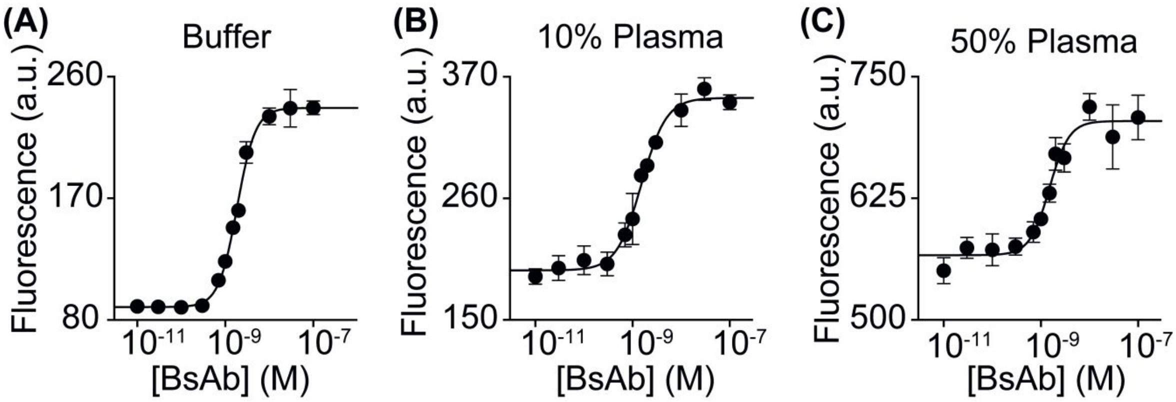

Figure S4 Binding curves obtained in 384 well plate at increasing concentrations of the BsAb (fit eq. (1)) in (A) buffer solution, (B) 10% and (C) 50% plasma solution. The experiments were performed in a 20 μL buffer solution (10 mM Na_2HPO_4, 137 mM NaCl, 2.7 mM KCl, pH 7.4), 10% and 50% plasma solutions each containing the reporter module (10 nM), the input module (30 nM), and Bispecific antibodies as indicated. The fluorescence signals were measured at 523 nm after 30 min information.

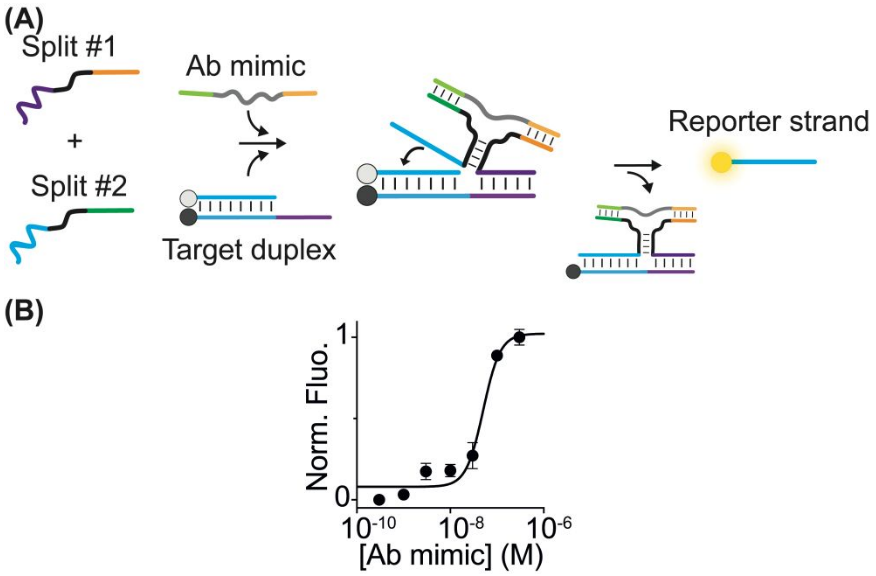

Figure S5 (A) Antibody-responsive DNA circuit designed to activate a strand displacement reaction upon the binding of a bivalent DNA strand (Ab mimic) to two Split #1 and #2 strands. (B) Binding curve obtained (fit eq. (2)) at increasing concentration of the Ab mimic strands using 60 nM of target duplex and 100 nM of Split #1 and #2. The experiments were performed in a 20 µL phosphate buffer solution (50 mM Na_2HPO_4, 150 mM NaCl, pH 7.0) and the fluorescence signals were measured at 680 nm.

Supplementary References

(1) Zadeh, J.N.; Steenberg, C.D.; Bois, J.S.; Wolfe, B.R.; Pierce, M.B.; Khan, A.R.; Dirks, R.M.; Pierce, N.A. NUPACK: Analysis and Design of Nucleic Acid Systems. *J. Comput. Chem.* **2011**, *32* (1), 170–173.

(2) Owczarzy, R.; Tataurov, A.V.; Wu,Y.; Manthey, J.A.; McQuisten, K.A.; Almabrazi, H.G.; Pedersen, K.F.; Lin, Y.; Garretson, J.; McEntaggart, N.O.; Sailor, C.A.; Dawson, R. B.; Peek, A.S. IDT SciTools: A Suite for Analysis and Design of Nucleic Acid Oligomers. *Nucleic. Acids. Res.* **2008**, *36* (Web Server issue), W163-169.
